# Determining the Targets of Fluopsin C Action on Gram-Negative and Gram-Positive Bacteria

**DOI:** 10.3389/fmicb.2020.01076

**Published:** 2020-06-04

**Authors:** Miguel Octavio Pérez Navarro, Guilherme Dilarri, Ane Stefano Simionato, Kathlen Grzegorczyk, Mickely Liuti Dealis, Barbara Gionco Cano, André Riedi Barazetti, Leandro Afonso, Andreas Lazaros Chryssafidis, Henrique Ferreira, Galdino Andrade

**Affiliations:** ^1^Microbial Ecology Laboratory, Department of Microbiology, Universidade Estadual de Londrina, Londrina, Brazil; ^2^Department of Biochemistry and Microbiology, Institute of Biosciences, Universidade Estadual Paulista, Rio Claro, Brazil; ^3^Department of Veterinary Medicine, Center of Agroveterinary Sciences, Universidade do Estado de Santa Catarina, Lages, Brazil

**Keywords:** bactericidal mechanism of action, *Xanthomonas*, KPC, MRSA, electronic microscopy, fluorescence microscopy

## Abstract

The antibiotic activity of metalloantibiotic compounds has been evaluated since the 90s, and many different modes of action were characterized. In the last decade, the effects of secondary metabolites produced by *Pseudomonas aeruginosa* LV strain, including a cupric compound identified as Fluopsin C, were tested against many pathogenic bacteria strains, proving their high antibiotic activity. In the present study, the bactericidal mechanisms of action of Fluopsin C and the semi-purified fraction F4A were elucidated. The results found in electron microscopy [scanning electron microscopy (SEM) and transmission electronic microscopy (TEM)] demonstrated that both Fluopsin C and F4A are affecting the cytoplasmatic membrane of Gram-positive and Gram-negative bacteria. These results were confirmed by fluorescence microscopy, where these bacteria presented permeabilization of their cytoplasmatic membranes after contact with the semi-purified fraction and pure compound. Using electronic and fluorescence microscopy, along with bacterial mutant strains with marked divisional septum, the membrane was defined as the primary target of Fluopsin C in the tested bacteria.

## Introduction

The development of metalloantibiotics as potential antimicrobial agents has been pursued since the 90s (Sekhon, 2010). The gene expression analysis of *Pseudomonas aeruginosa* LV strain cultured in the presence of copper chloride suggested that the intracellular excess of copper forms a compound with antimicrobial activity (Gionco et al., 2017). Other studies demonstrated that a specific fraction extracted from the supernatant of *P. aeruginosa* LV strain culture (F4A) presents high microbicidal activity against many pathogenic bacteria (de Oliveira et al., 2011, 2016; Cardozo et al., 2013; Murate et al., 2015; Kerbauy et al., 2016; Munhoz et al., 2017). F4A also showed strong antimicrobial effect against planktonic cells and biofilm formation of sixty-nine MDR 70 isolates, including *Klebsiella pneumoniae Kpn*-19 (Kerbauy et al., 2016). One of the main bioactive compounds produced by *P. aeruginosa* LV strain is a metalloantibiotic (organocopper compound), which is promising to become a new antibiotic in the control of infections caused by MDR bacteria. This metalloantibiotic was identified as Fluopsin C (YC 73), and it is a secondary metabolite produced by *Pseudomonas* spp. and *Streptomyces* sp., with high antibacterial, antifungal, and antitumor activities (Itoh et al., 1970; Otsuka et al., 1972; Ma et al., 2013). However, little is known about the mechanisms of action of Fluopsin C on Gram-negative and Gram-positive bacteria.

In previous studies, it was observed that pathogenic bacteria treated with the semi-purified fraction F4A fraction or Fluopsin C presented complete disruption of cell membrane and frequent presence of cells that stopped septation (Vasconcellos et al., 2014; Murate et al., 2015; de Oliveira et al., 2016; Kerbauy et al., 2016; Munhoz et al., 2017; Navarro et al., 2019). However, to the best of our knowledge, the major mechanism of action of these compounds has not yet been defined. The determination of the mechanisms of action of an antimicrobial compound is essential for defining how the bacterial damage happens, and which possible mechanisms of resistance may arise in the future. Another advantage is the determination of the time needed by the antimicrobial agent to inhibit bacterial development, depending on the mechanism of action.

Therefore, the objective of this study was to determine the action target of Fluopsin C using *Xanthomonas citri* subsp. *citri* and *Klebsiella pneumoniae* Kpn*-*19 as Gram-negative models and *Staphylococcus aureus* ATCC 29213 and *Enterococcus faecium* ATCC 6569 as Gram-positive bacteria.

## Materials and Methods

### Bacterial Strain and Growth Conditions

*Xanthomonas citri* subsp. *citri* strain (*Xcc*) used for the membrane integrity analyses was the isolate 306 (IBSBF 1594) (Schaad et al., 2006). The mutant strain *X. citri amy*:pPM2a-zapA, expressing GFP-ZapA that labels the divisional septum, was used to investigate the interference on bacterial cell division (Martins et al., 2010). Both *X. citri* strains were cultivated in NYG/NYG-agar medium (nitrogen-yeast-glycerol: 5 g/L of peptone, 3 g/L of yeast extract, 2% glycerol; for solid medium bacterial agar was added to 15 g/L) at 28°C for 24 h, in and orbital shaker at 200 rpm. *K. pneumoniae Kpn*-19 was isolated from patients attended at the University Hospital of Londrina, PR, Brazil, and it is deposited at the microbial collection of Microbiology Laboratory of the same hospital (Kerbauy et al., 2016). *K. pneumoniae Kpn*-19, *E. faecium* ATCC 6569, and *S. aureus* ATCC 29213, were cultured in MH/MH (Müeller–Hilton medium) at 37°C.

### Effect of F4A Fraction and Fluopsin C on the Ultra-Structure of Gram-Negative and Gram-Positive Bacteria

The F4A fraction and Fluopsin C used in this study were obtained according to procedures described by Munhoz et al. (2017) and Navarro et al. (2019), respectively. The F4A contains basically four compounds (mg/L): phenazine-1-carboxylic acid (14 mg), phenazine-carboxamide (9 mg), indol-3-one (1 mg), and Fluopsin C (6 mg) (Bedoya et al., 2019). IC_90_ was used in the following assays for enhancing the analyses. The majority of dead bacteria reflect the antibiotic effect of the tested compounds, but the remaining organisms present different stages of cell destruction, allowing the detailed observation of their mechanisms of action. For the scanning electron microscopy assay (SEM), bacterial suspensions (10^10^ CFU) of each strain were incubated for 60 min with and without IC_90_ concentrations of F4A (0.25 μg mL^–1^ for *X. citri*) (Murate et al., 2015) and Fluopsin C (2 μg mL^–1^ for *K. pneumoniae*, 1 μg mL^–1^ for *E. faecium*, and 0.5 μg mL^–1^ for *S. aureus*) (Navarro et al., 2019) were spotted onto poly-L-lysine-coated slides and stored at 28°C for 1 h for drying. The slides were fixed with a solution containing 2% paraformaldehyde and 2.5% glutaraldehyde in 0.1 M sodium cacodylate buffer (pH 7.0) for 12 h. After that, the slides were washed with 0.1 M sodium cacodylate buffer (pH 7.0) and post-fixed in a solution of 1% OsO_4_ for 2 h. The samples were dehydrated in ethanol at concentrations of 70, 80, 90, and 100% and then dried by critical point in CO_2_ (BALTEC CPD 030 Critical Point Dryer). After drying, the slides were coated with gold (BALTEC SDC 050 Sputter Coater) and visualized under a scanning electron microscope (FEI Quanta 200).

For the transmission electronic microscopy (TEM) assay, microorganisms were incubated with and without IC_90_ concentrations of F4A (0.25 μg mL^–1^ for *X. citri* 306) and Fluopsin C (2 μg mL^–1^ for *K. pneumoniae* and 0.5 μg mL^–1^ for *S. aureus*). The samples were centrifuged at 4,000 rpm for 5 min. The pellets were resuspended, washed with PBS, centrifuged again, and fixed as described above. After dehydration in a series of ethanol, the material was included in Araldite. Ultra-thin cuts of 60–70 nm (Leica ULTRACUT Ultramicrotome) were made in the block and contrasted with 2% uranyl acetate for 15 min and lead citrate for 20 min and observed under the transmission electron microscope (FEI Tecnai 12).

### Membrane Permeability and Cell Division Disruption Assays

Cells of *E. faecium*, *S. aureus*, and *X. citri* (10^5^ CFU) were incubated in 0.1 mL of the respective culture medium for 15 min containing IC_90_ concentrations of F4A (0.25 μg mL^–1^ for *X. citri*) and Fluopsin C (1 μg mL^–1^ for *E. faecium* and 0.5 μg mL^–1^ for *S. aureus*). Right after this incubation period, 0.9 mL of each respective culture medium was added to the reaction tubes in order to stop the treatments. Tubes were centrifuged at 3,000 × *g* for 30 s, the supernatant was discarded, and the cells were resuspended in 100 μL of sterile saline (0.9% NaCl). Membrane integrity was verified by staining the cells with DAPI and propidium iodide (IP), according to the manufacturer instructions (LIVE/DEAD *Bac*Light Bacterial Viability Kit; Thermo Fisher Scientific L7012). DAPI (4′,6-diamidino-2-phenylin-dole) is a DNA-specific probe, a fluorescent dye that naturally enters the cells, regardless of whether they are alive or with corrupted membranes. DAPI stains the genetic material/bacterial nucleoid (Kapuscinski, 1995). With the nucleoid stained, it is possible to observe any anomalous segregation of the bacterial chromosome before or after cell division. Ten microliter of each cell suspension was placed on microscope slides covered with 1% agarose in saline for microscopic observation (Martins et al., 2010). To generate the positive controls for membrane permeability, *X. citri* cells were heated for 2 min at 55°C (Sumares et al., 2016; Savietto et al., 2018), and Gram-positive bacteria were exposed to Nisin (SIGMA N5764) (Król et al., 2015). DAPI labeling was also applied to investigate possible chromosome segregation defects, such as misplaced bacterial nucleoids, and the presence of anucleated cells.

For evaluating cell division, 100 μL of *X. citri amy*:pPM2a-*zapA* culture, containing 10^5^ cells, were treated with F4A or fluopsin C at their IC90 concentration (0.25 μg mL^–1^ for both compounds) or 1% DMSO (vehicle control) for 15 min at 30°C. After the incubation period, the volume was increased to 1 mL to stop the reaction, and tubes were centrifuged at 3,000 × *g* for 30 s. The supernatant was discarded, and cells were resuspended with 100 μL of sterile saline (0.9% NaCl). Ten microliter of the cell suspension was put onto slides covered with 1% agarose 1% in saline for microscopic observation (Martins et al., 2010).

All samples were examined by fluorescence microscopy using an Olympus BX-61 optical microscope, equipped with a Fluorescence UIS2 optical system (DAPI excitation 372 nm/emission 456 nm; Tx red excitation 596 nm/emission 620 nm; GFP excitation 475 nm/emission 509 nm). Images were captured using a monochromatic camera Orca-Flash 2.8 (Hamamatsu, Japan), with the software CellSens v. 11 (Olympus). All the experiments were performed in triplicates with a minimum of *n* = 200 cells per experiment.

## Results

### Effect of F4A Fraction and Fluopsin C on the Ultra-Structure of Gram-Negative and Gram-Positive Bacteria

**FIGURE 1:**
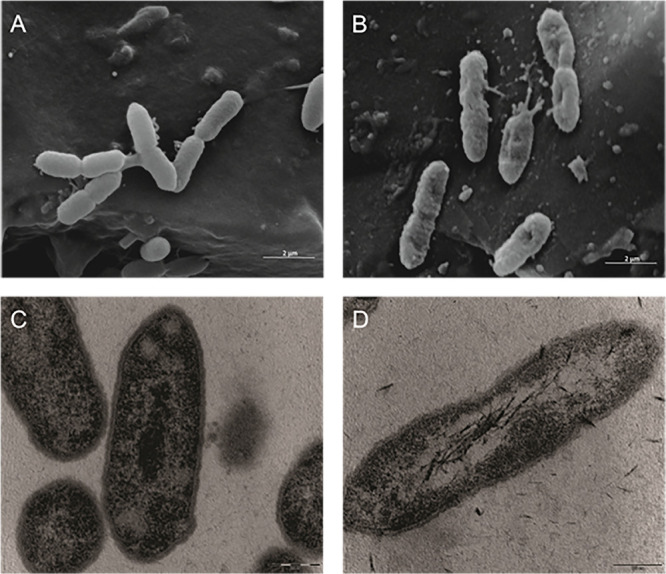
Effect of the F4A fraction on the ultra-structure of the Gram-negative bacteria *Xanthomonas citri* subsp. *citri*, strain 306 strain. Scanning electron microscopy: **(A)** control (non-treated) (20,000×); **(B)** cells treated with F4A fraction (20,000×). Transmission electron microscopy **(C)** control (non-treated) (46,000×); **(D)** cells treated with F4A fraction (46,000).

Both the SEM analysis revealed that untreated *X. citri* subsp. *citri* 306 strain cells did not present any morphological changes (Figure 1A) when compared to the treated cells, where Fluopsin C disrupted the membrane and cells appeared shrunken and rough compared to the control (Figure 1B). At the TEM, it was observed that the cell wall and the cytoplasmic membrane of non-treated *X. citri* appeared to be intact (Figure 1C), while treated cells presented modified cell shape and morphology (Figure 1D), being elongated and with affected cell wall and cytoplasmic membrane, becoming indistinct.

**FIGURE 2:**
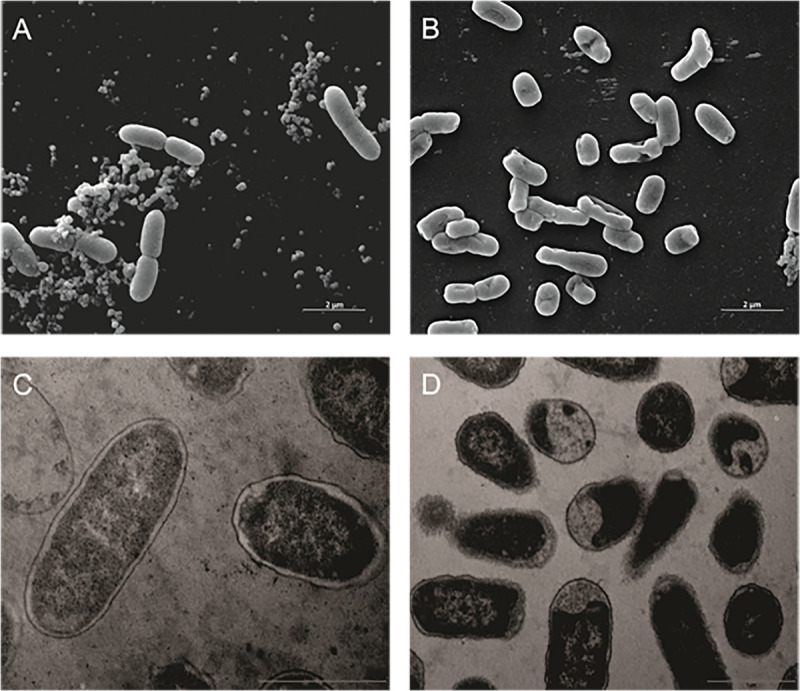
Effect of the antibiotic activity of Fluopsin C on the ultra-structure of the Gram-negative bacteria *Klebsiella pneumoniae Kpn*-19. Scanning electron microscopy: **(A)** control (non-treated) (10,000×); **(B)** cells treated with Fluopsin C (10,000×). Transmission electron microscopy **(C)** control (non-treated) (26,500×); **(D)** cells treated with Fluopsin C (18,500×).

The same alterations in cell ultra-structure were observed in other Gram-negative bacteria. In the SEM, *K. pneumoniae Kpn*-19 strain control cells were intact (Figure 2A) and treated cells had their cell membranes and walls disrupted (Figure 2B). Similar effects were observed by TEM, where the control cells were intact (Figure 2C) and treated cells presented significant changes in the cell membrane and wall, as well as in the cytoplasm (Figure 2D).

**FIGURE 3:**
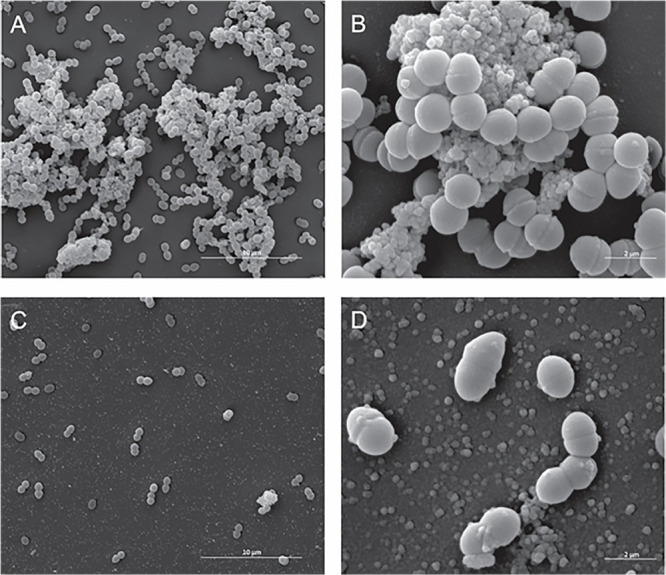
Effect of the antibiotic activity of Fluopsin C on the ultra-structural of the Gram-positive bacteria *Enterococcus faecium* strain ATCC 6569. Scanning electron microscopy: **(A,B)** control (non-treated) – **(A)** 8,000× and **(B)** (40,000×); **(C,D)** cells treated with Fluopsin C – **(C)** (8,000×) and **(D)** (40,000×).

With Gram-positive bacteria, SEM analysis did not unveil marked differences between treated and untreated *Enterococcus faecalis* ATCC 6569 strain cells (Figures 3A,B), but the number of cells decreased severely when Fluopsin C was present (Figures 3C,D).

**FIGURE 4:**
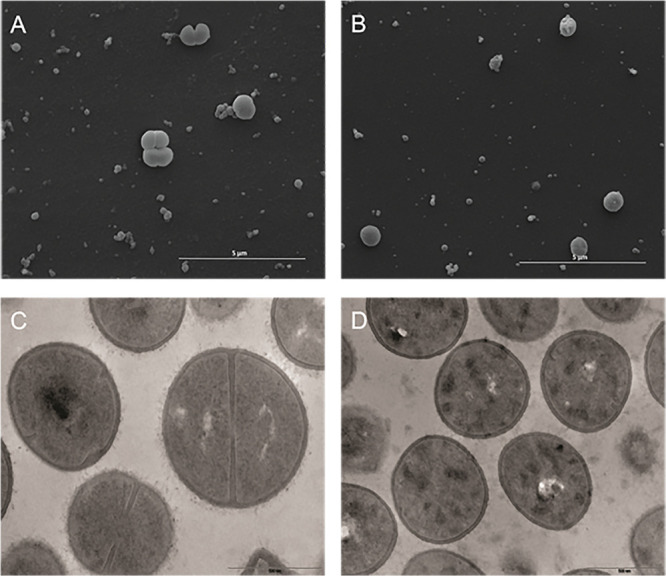
Effect of the antibiotic activity of Fluopsin C on the Gram-positive bacteria *Staphylococcus aureus* strain MRSA N315. Scanning electron microscopy: **(A)** control (non-treated) (10,000×); **(B)** cells treated with fluopsin (10,000×) (46,000×). Transmission electron microscopy **(C)** control (non-treated) (46,000×); **(D)** cells treated with Fluopsin C (37,000×).

On the other hand, *S. aureus* MRSA N315 strain–treated cells had overt changes in their cell membrane and wall when compared with the controls (Figures 4A,B). Fluopsin C exposed cells apparently lost their cell wall and presented heterogeneous and electron dense cytoplasm when compared to untreated cells (Figures 4C,D).

### Membrane Permeability Assay

**FIGURE 5:**
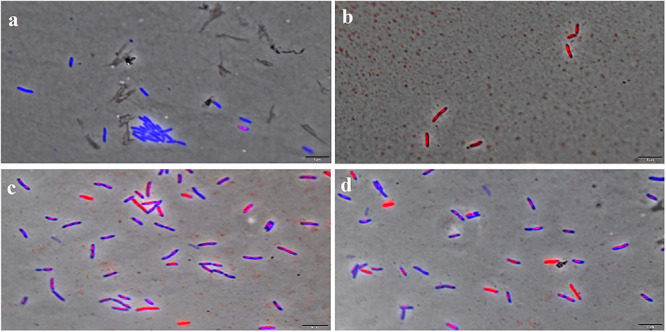
F4A and Fluopsin C permeabilized the membrane of *X. citri* subsp. *citri* strain 306. Cells were exposed to the compounds at their respective IC_90_ values for 15 min and stained with DAPI/IP before microscope observation. Cells with intact membrane are shown in blue, while cells with disrupted membrane are in red. **(A)** non-treated cells; **(B)** positive control, cells with membrane permeabilized by temperature stress; **(C)** cells treated with Fluopsin C; **(D)** cells treated with F4A fraction. Images are the overlay of phase-contrast microscopy and fluorescence. The filters Tx Red and DAPI Blue were applied together and used to visualize IP and DAPI. The bars correspond to 5 μm; magnification of 100X.

**FIGURE 6:**
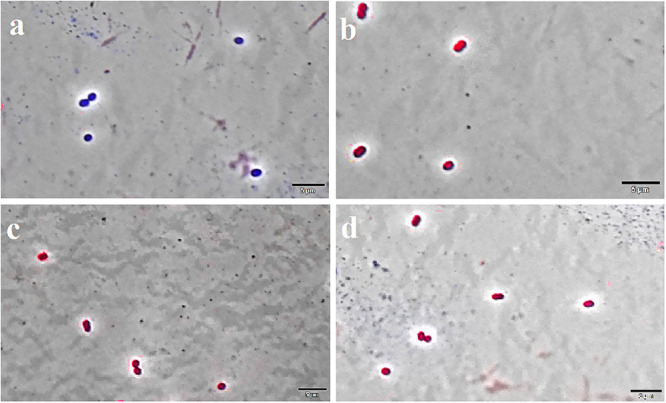
F4A and Fluopsin C permeabilized the membrane of *E. faecium* ATCC 6569. Cells were exposed to the compounds at their respective IC_90_ values for 15 min, and after staining with DAPI/IP before microscope observation. Cells with intact membrane are shown in blue, while cells with disrupted membrane are in red. **(A)** non-treated cells; **(B)** positive control, cells treated with nisin at 2.5 μg/mL; **(C)** cells treated with Fluopsin C; **(D)** cells treated with F4A fraction. Images are the overlay of phase-contrast microscopy and fluorescence. The filters Tx Red and DAPI Blue were applied together and used to visualize IP and DAPI. The bars correspond to 5 μm; magnification of 100X.

**FIGURE 7:**
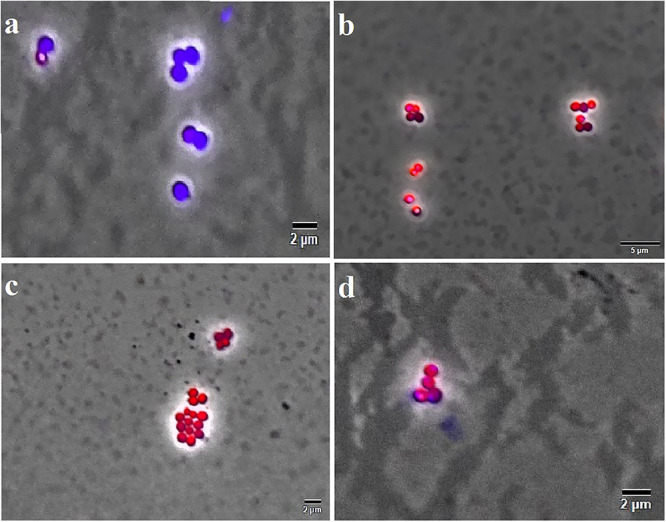
F4A and Fluopsin C permeabilized the membrane of *S. aureus* strain ATCC 29213. Cells were exposed to the compounds at their respective IC_90_ values for 15 min, and after staining with DAPI/IP before microscope observation. Cells with intact membrane are shown in blue, while cells with disrupted membrane are in red. **(A)** non-treated cells; **(B)** positive control, cells treated with nisin at 2.5 μg/mL; **(C)** cells treated with Fluopsin C; **(D)** cells treated with F4A fraction. Images are the overlay of phase-contrast microscopy and fluorescence. The filters Tx Red and DAPI Blue were applied together and used to visualize IP and DAPI. The bars correspond to 5 μm; magnification of 100X.

The Live/Dead test showed that F4A fraction and Fluopsin C affected the cell membrane of *X. citri* after 15 min of exposure, causing serious damage and increasing its permeability (Figure 5). The F4A fraction and Fluopsin C also affected the cell membranes of *E. faecium* ATCC 6569 (Figure 6) and *S. aureus* ATCC 29213 (Figure 7), as reflected by the increased permeability to the nucleoid dye propidium IP compared to the control untreated.

**FIGURE 8:**
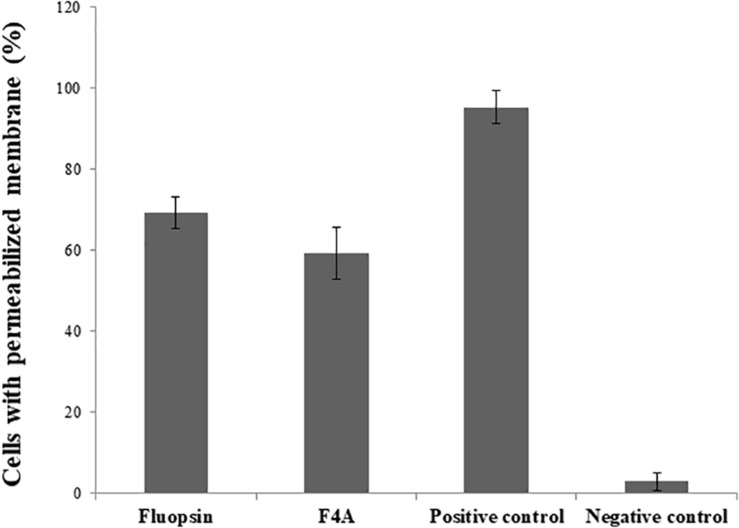
Percentage of *Xanthomonas citri* subsp. *citri* cells with permeabilized membrane caused by the exposure to F4A and fluopsin. Negative control consists of untreated cells, and positive control consists of cells permeabilized by heat stress.

No difference was observed in the activities of both F4A and Fluopsin C, where approximately 60% of *X. citri* cells presented a similar extent of membrane disruption when compared to the positive control (heat shock; Figure 8). The results demonstrate that the cell membrane is one of the targets of F4A and Fluopsin C.

**FIGURE 9:**
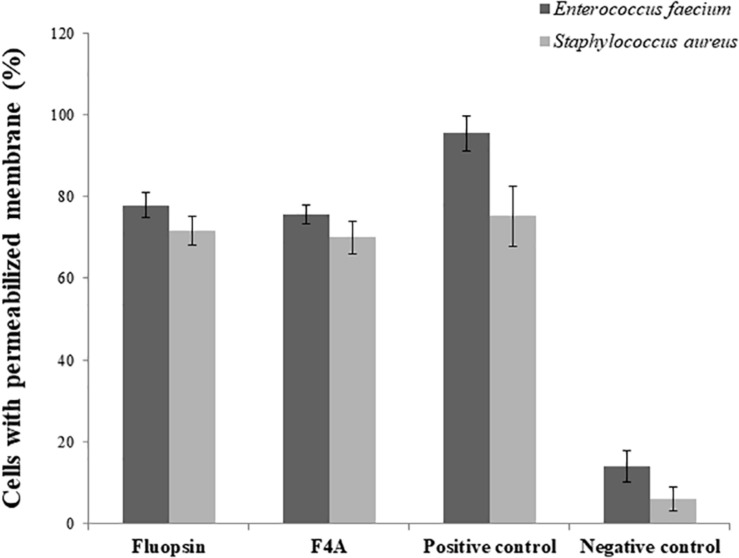
Percentage of *Enterococcus faecium* ATCC 6569 and *Staphylococcus aureus* ATCC 29213 cells with permeabilized membrane caused by the exposure to F4A and fluopsin. Negative control consists of untreated cells, and positive control consists of cells permeabilized by nisin.

There was little difference in the number of cells with disrupted membrane treated with F4A and Fluopsin C. Both compounds affected more than 70% of the *E. faecium* ATCC 6569 and *S. aureus* ATCC 29213 cells (Figure 9), and results were very close to the positive control (treated with Nisin). Therefore, the results suggested that the plasmatic membrane is one of the targets of F4A and Fluopsin C for *E. faecium* ATCC 6569 and *S. aureus* ATCC 29213 as well. F4A and Fluopsin C were more effective against *S. aureus* ATCC 29213, with very similar results to the cells treated with Nisin.

### Evaluation of Cell Septum Disruption Analyses

**FIGURE 10:**
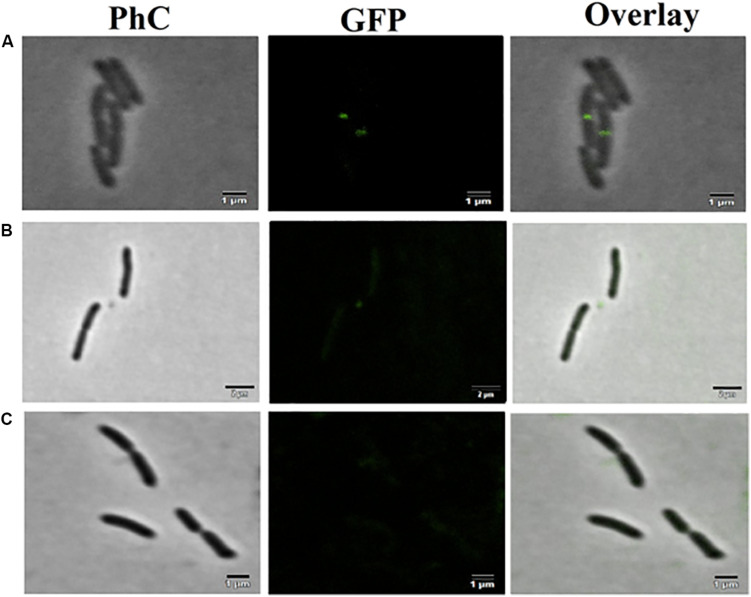
F4A and fluopsin perturbed the divisional septum of *X. citri.* The mutant strain *X. citri* amy:pPM2a-zapA, labeled for the divisional septum, was exposed to F4A or fluopsin at their IC_90_ values for 15 min, and after being analyzed by fluorescent microscopy. **(A)** untreated control; **(B)** cells treated with fluopsin; **(C)** cells treated with F4A. The divisional septum corresponds to the green bar perpendicular to the long axis of the rods. The bar corresponds to 1 μm; magnification of 100X.

**FIGURE 11:**
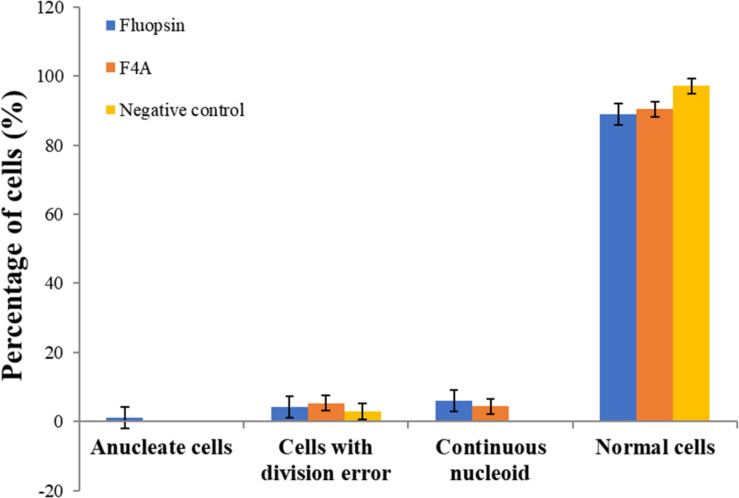
F4A and Fluopsin C did not interfere with chromosome segregation and cell division in *X. citri*. Cells of *X. citri* were exposed to F4A or Fluopsin C at their IC_90_ values for 15 min and stained with DAPI before microscope observation. The percentage of cells showing division and chromosome segregations errors, such as filamentation, anucleate cells, and/or continuous nucleoid were determined by comparison with the negative control (untreated cells). Experiments were performed in triplicate (200 cells were analyzed per experiment; *n* = 200).

The mutant strain *X*. *citri amy*:pPM2a-*zapA*, labeled for the divisional septum, when treated with F4A or Fluopsin C, indicated that both compounds can affect the cellular division by interfering in the Z-ring of *X*. *citri* (Figure 10). Untreated *X*. *citri amy*:pPM2a-*zapA* displays a fluorescent bar, the Z-ring, perpendicular to the long axis of the rods for the cells that are preparing to divide (Figure 10A). After exposure to both compounds for 15 min, the Z-ring was completely dissolved and could not be detected any longer (Figures 10B,C). However, subsequent morphological examination of wild-type *X. citri* by phase-contrast microscopy, and analysis of nucleoid distribution using DAPI, did not evidence any increase in the number of abnormal cells, i.e., filaments and cells with increased size, minicells, and dividing cells showing septal constriction and continuous nucleoid at the same time (*p* < 0.5) (Figure 11). *X*. *citri* did not show any increase in cell size after treatment with F4A or Fluopsin C (Table 1).

**TABLE 1:** Morphological analyses of cell size of *X. citri* subsp. *citri.*

	**Longer cell (μm)**	**Shorter cell (μm)**	**Mean (μm)**	*
Negative control	3.45	1.05	1.66	–
F4A	3.30	0.96	1.80	n.s.
Fluopsin	3.80	0.95	1.90	n.s.

*Means of hundred cells. Non-significant (n.s.) (*p* < 0.5) when compared with treated cells and non-treated cells.

## Discussion

F4A and Fluopsin C generated the same effect on *Xcc* 306 that was observed earlier by de Oliveira et al. (2011), where the cell membrane and wall were completely disrupted. The same action was observed in *Kpn*-19 strain treated with Fluopsin C (Navarro et al., 2019), suggesting that the compound acts in the same way in different Gram-negative genera.

Additionally, the antibiotic activity of Fluopsin C was tested in two different genera of Gram-positive bacteria, but the effects on cell ultrastructural were not the same in *E. faecium* ATCC 6569 and MRSA N315. In *E. faecium* ATCC 6569 strain, despite the decrease in cell numbers after treatment with Fluopsin C, no damage was detected in its cell membrane and wall. On the other hand, treated MRSA N315 strain bacteria presented the same changes on cell membrane and wall that was observed in Gram-negative bacteria, with cell disruption and changes in the cytoplasm. Similar activity was reported previously (Navarro et al., 2019).

Different authors observed the effect of F4A on cell morphology by scanning and transmission electron microscopy of *X. citri* subsp. *citri* (de Oliveira et al., 2011), other Gram-negative bacteria (Lopes et al., 2012; Murate et al., 2015; Kerbauy et al., 2016; Munhoz et al., 2017), and Gram-positive bacteria (Cardozo et al., 2013). In the present study, the complete disruption of the cell membrane was verified after 15 min of exposure to F4A. Also, cells remained attached during duplication (de Oliveira et al., 2011), suggesting that F4A may inhibit the cell septation process.

Fluopsin C pure or present in F4A fraction inhibited *E. faecium* ATCC 6569 and *S. aureus* ATCC 29213 due to the disarrangement of cell membrane structure, leading both strains to death after 15 min of treatment, suggesting that both compounds also act on the cell membrane structure of Gram-positive bacteria; this result corroborated and clarified the findings of Navarro et al. (2019).

The compounds did not impair significantly in the cell division process, as there was no disarrange in the genetic material or in the chromosomal segregation process. However, F4A and Fluopsin C were able to disrupt *X. citri* divisional septum. This effect was probably secondary to the breakdown of the membrane potential. The membrane potential is required to keep FtsZ protein in the correct localization on the membrane, and it corroborates the observed effect (Strahl and Hamoen, 2010). It is possible to conclude that the primary target of Fluopsin C is the cell membrane, and the divisional septum becomes delocalized because of the interference with the membrane potential. Other targets may be involved, and further studies should be carried out.

## Conclusion

Electronic microscopic analysis demonstrated that F4A and Fluopsin C are affecting the cytoplasmatic membrane of Gram-positive and Gram-negative bacteria used in this study. This corroborates with the results of fluorescence microscopy, where Gram-positive and Gram-negative bacteria presented permeabilization of their cytoplasmatic membranes after contact with F4A and Fluopsin C.

The experiment using the mutant *X*. *citri amy*:pPM2a-*zapA* showed that the divisional ring is affected by both antimicrobial compounds, but it was concluded that this is a secondary effect. The cytoplasmatic membrane damage leads to dissolution of the divisional septum, by the disturbance of the cellular membrane potential. Morphological analysis of the cells also confirmed it, since the treatment with the antimicrobial compounds did not lead to any significant increase in the number of aberrant cells. Overall, it can be concluded that the cytoplasmatic membrane is the primary target of F4A and Fluopsin C.

The effect on cell septation was not completely understood, and further studies need to be carried out. The cells may pause the division process many times, especially in Gram-negative bacteria, but it was also observed in *S. aureus*.

## Data Availability Statement

The datasets generated for this study are available on request to the corresponding author.

## Author Contributions

GA, HF, and MN conceived the study and designed the experimental procedures. MN, AS, KG, MD, BC, AB, and LA carried out the experiments. MN, AS, GD, AC, and GA analyzed the data. GD and HF contributed reagents and materials. MN, GA, HF, and AC wrote this manuscript. GA supervised the project.

## Conflict of Interest

The authors declare that the research was conducted in the absence of any commercial or financial relationships that could be construed as a potential conflict of interest.
