# Corrigendum: Determining the Targets of Fluopsin C Action on Gram-Negative and Gram-Positive Bacteria

**DOI:** 10.3389/fmicb.2020.574002

**Published:** 2020-10-06

**Authors:** Miguel Octavio Pérez Navarro, Guilherme Dilarri, Ane Stefano Simionato, Kathlen Grzegorczyk, Mickely Liuti Dealis, Barbara Gionco Cano, André Riedi Barazetti, Leandro Afonso, Andreas Lazaros Chryssafidis, Henrique Ferreira, Galdino Andrade

**Affiliations:** ^1^Microbial Ecology Laboratory, Department of Microbiology, Universidade Estadual de Londrina, Londrina, Brazil; ^2^Department of Biochemistry and Microbiology, Institute of Biosciences, Universidade Estadual Paulista, Rio Claro, Brazil; ^3^Department of Veterinary Medicine, Center of Agroveterinary Sciences, Universidade Do Estado de Santa Catarina, Lages, Brazil

**Keywords:** bactericidal mechanism of action, *Xanthomonas*, KPC, MRSA, electronic microscopy, fluorescence microscopy

In the original article, we neglected to include the funder São Paulo Research Foundation, FAPESP 2017/50216-0 to Henrique Ferreira.

The authors apologize for this error and state that this does not change the scientific conclusions of the article in any way. The original article has been updated.

